# Microbial Inoculation Enhances Growth and Physiological Traits of Tissue Cultured *Panicum turgidum* Forssk. Plantlets During Acclimatization

**DOI:** 10.3390/plants15152252

**Published:** 2026-07-23

**Authors:** Muhammad M. Habib, Yaser Hassan Dewir, Thobayet S. Alshahrani, Jahangir A. Malik, Basharat A. Dar, Abdulaziz A. Al-Qarawi

**Affiliations:** Department of Plant Production, College of Food and Agricultural Sciences, King Saud University, Riyadh 11451, Saudi Arabia

**Keywords:** acclimatization, *Bacillus subtilis*, desert grass, *Rhizophagus fasciculatus*, tissue culture, *Trichoderma harzianum*

## Abstract

*Panicum turgidum* is an important keystone forage grass crucial for ecosystem stability in arid and semi-arid rangelands of Asia and Africa. This study aimed to investigate the use of beneficial bioinoculants for enhancing the growth and acclimatization of micropropagated desert grass under controlled environmental conditions. Tissue-cultured plants were transferred to pots with a sand–soil mixture (1:1, *v*/*v*) and kept in a temperature and light-controlled environment (25 ± 2 °C, 100 µmol m^−2^·s^−1^, and 16/8 light/dark photoperiod) for 10 weeks. We used a factorial approach to investigate the effects of the arbuscular mycorrhizal fungus (AMF) *Rhizophagus fasciculatus*, *Trichoderma harzianum*, and *Bacillus subtilis* on transplanted desert grass plants. Two AMF levels control (AMF, 5%; *w*/*w*) or without AMF (NAMF); and three microbial inoculation treatments (*T. harzianum*, *B. subtilis*, and a combination of *T. harzianum* + *B. subtilis*) were employed. Microscopic investigation indicated the extent of AMF colonization in the roots of micropropagated *P. turgidum* plantlets during acclimatization. Growth parameters with shoots and roots, chlorophyll and carotenoid content, and nitrogen uptake were all improved in bioinoculants-treated plants. Under non-AMF conditions, co-inoculation with *T. harzianum* and *B. subtilis* resulted in the highest biomass and root traits, whereas under AMF conditions, *T. harzianum* alone or with AMF was generally most effective. In the plants treated with the combination of inoculants, heatmap correlation, principal component analysis, and hierarchical clustering demonstrated positive association between growth and physiological traits and the absorption of mineral nutrients, although these associations varied among treatment combinations. These findings highlight the potential of beneficial bioinoculants to improve the growth and acclimatization of micropropagated desert grass plants, providing a foundation for tailored bioinoculant compositions towards the rehabilitation of degraded grazing lands.

## 1. Introduction

The arid and semi-arid regions are among the most fragile terrestrial ecosystems and are experiencing heightened desertification and ecosystem degradation due to prolonged drought, high temperatures, soil salinity, nutrient-poor soils, and continuous human influence [1,2]. Ultimately, these degraded ecosystems threaten the existence of vegetation composition and the livelihoods of communities reliant on these ecosystems, making immediate action, through the establishment of native perennial species against this global problem, a key ecological priority for improving ecosystem resilience and sustainability [3]. *Panicum turgidum* Forssk. (Poaceae) is a C4 perennial desert grass distributed in the arid regions of Africa, especially around the Nile and the Mediterranean coastal strip, extending through the Arabian Peninsula, including Saudi Arabia, to Southwest Asia [4,5]. This grass is considered an excellent forage grass and an important keystone species for rangeland rehabilitation due to its thick fibrous root system, which contributes to sand and soil binding [6,7]. It grows in dense bushes and is valued for its forage with 25% carbohydrate, 13% crude protein, 18% crude fiber, and 2.34% oxalates and minerals, including potassium, sodium, magnesium, calcium, and phosphorus [6,8]. However, despite the ecological and economic importance of *P. turgidum*, its large-scale restoration is constrained by poor natural regeneration, due to low seed germination and prolonged seed dormancy, which limits conventional propagation and necessitates tissue culture for large-scale production for rehabilitation programs in degraded arid lands.

An efficient tissue culture technique offers an effective strategy necessary for the successful application of biotechnology for the large-scale production of plantlets and their subsequent acclimatization independent of seed availability [9]. The micropropagated plants are utilized for eco-rehabilitation and to restore the degraded desert ecosystem. However, the successful establishment of micropropagated plants under ex vitro and field conditions remains a major challenge. Plantlets regenerated in vitro typically possess poorly developed cuticles, non-functional stomata, limited root systems, and reduced microbial associations, making them highly susceptible to transplant shock, water deficit, and oxidative stress during acclimatization to *ex vitro* conditions [10]. Therefore, certain acclimation procedures are required when moving in vitro regenerated plants to ex vitro environments, as they are transferred from controlled light, temperature, and relative humidity conditions to high light, high temperature, and low humidity conditions. Micropropagated plants typically experience transplantation stress [11,12]. As a result, many plants have been propagated under photoautotrophic conditions, which include using ventilated culture vessels, growing plantlets under increased light and carbon dioxide conditions, and reducing the amount of sucrose in the medium. These conditions allow plants to grow properly, resulting in profuse rooting and involving sustainable photosynthetic conditions [13,14]. As an alternative biological approach, several researchers have examined the impact of colonizing micropropagated plantlets with *Rhizophagus* spp. (arbuscular mycorrhizal fungi or AMF), *Bacillus* spp. (plant growth-promoting rhizobacteria or PGPR), and *Trichoderma* spp. (free-living fungi). This is known as the biological method of acclimatization of tissue culture plants. *Azospirillum*, *Bacillus*, *Gigaspora*, *Glomus*, *Pseudomonas*, *Rhizophagus*, *Trichoderma*, and other soil microorganisms have been employed to promote plant growth and improve tolerance to abiotic stresses [15,16,17,18,19,20]. By encouraging the growth and development of shoots and roots, improving nutrient uptake, and inducing systemic resistance, the application of *Bacillus*, *Gigaspora*, *Glomus*, and *Pseudomonas* has been specifically investigated to improve the acclimatization and adaptation of in vitro regenerated plants, thereby facilitating a more successful transition to ex vitro conditions [21,22,23,24,25,26,27,28,29,30,31]. These characteristics work in concert to facilitate the successful ex vitro establishment and overall growth enhancement of tissue culture-derived plants, thereby supporting their application in the biological hardening of plants.

The beneficial effects of AMF, *Trichoderma*, and *Bacillus* have been extensively studied in crops, horticultural species, and several micropropagated plants [15,32,33]. However, the combined application of these bioagents in biological hardening and establishment of native plants is reported for *Melia volkensii* plantlets [34] and remains largely unexplored for the establishment of native grasses under arid conditions. In particular, there is no information available regarding the interactive effects of these bioagents on the acclimatization and early establishment of micropropagated *P. turgidum*.

The current study hypothesized that inoculation with *Rhizophagus fasciculatus*, *Trichoderma harzianum*, and *Bacillus subtilis*, either individually or in combination, would enhance the acclimatization and ex vitro establishment of micropropagated *P. turgidum* by improving plant growth, physiological performance, nutrient acquisition, and stress tolerance. Therefore, in this study, we evaluated the individual and combined effects of these three beneficial microorganisms on the growth, physiological attributes, mineral nutrition, and survival of tissue culture-derived *P. turgidum* during acclimatization. This will help us to develop an efficient strategy for the biological hardening of ecologically important native plants, which is a way forward for the large-scale restoration of degraded arid rangelands.

## 2. Results

### 2.1. Microbial Colonization

The effects of arbuscular mycorrhizal fungus (AMF) treatment (5% *w*/*w*, *R. fasiculatus*) or without AMF (NAMF) on the growth, physiology, and nutritional parameters of desert grass plants were investigated in a factorial design. The other factors included microbial inoculation, including (1) control (AMF/NAMF), (2) treatment with *T. harzianum*, (3) treatment with *B. subtilis*, and (4) treatment with a combination of *T. harzianum* + *B. subtilis* (Figure 1). After ten weeks of acclimatization, the roots of desert grass tissue culture plants were collected and examined for colonization and sporulation on inoculants with host plants. Intact spores, intercellular hyphae, arbuscules, vesicles, extracellular hyphae, coiled hyphae, and germinating spores were all visible under a microscope (Figure 2). Compared with *T. harzianum* + *R. fasciculatus*, *B. subtilis* + *R. fasciculatus*, and *T. harzianum* + *B. subtilis* + *R. fasciculatus*, the spore count analysis showed 108 spores per 100 g dry soil when plants were inoculated with *R. fasciculatus* (Figure 1a). Similarly, compared to other treatments, *T. harzianum* + *B. subtilis* + *R. fasciculatus* showed 67% mycelium growth (Figure 1b) and 48% arbuscule production (Figure 1c).

### 2.2. Plant Growth Parameters

The growth parameters of plants were evaluated ten weeks after transplantation under various treatments; the results are shown in Figure 3. Under Non-AMF conditions, *T. harzianum* increased leaf area, shoot length, stem diameter, and shoot dry weight compared with the uninoculated control, whereas *B. subtilis* primarily enhanced shoot fresh weight and produced intermediate responses for the remaining traits. Co-inoculation with *T. harzianum* and *B. subtilis* resulted in the highest leaf area, fresh and dry weights, stem diameter, shoot fresh and dry weights, as well as shoot length, although leaf and node numbers were not significantly affected. Under AMF conditions, the uninoculated control produced the highest leaf and node numbers and maintained high shoot fresh weight. The addition of *T. harzianum* increased leaf area and shoot length, while *B. subtilis* produced intermediate responses for most traits. Co-inoculation with *T. harzianum* and *B. subtilis* did not consistently outperform the individual inoculations, although shoot fresh weight remained among the highest values. Plants treated with *T. harzianum* + *B. subtilis* resulted in a significant improvement in plant growth parameters. When compared to the control (NAMF and AMF), the leaf area (8.38 cm^2^), leaf fresh weight (0.27 g), leaf dry weight (0.05 g), shoot length (71.21 cm), stem diameter (1.85 mm), shoot fresh weight (2.10 g), and shoot dry weight (0.49 g) were all significantly higher. However, the plants treated with AMF had more leaves (13.4) and nodes (8.4) per plant. These results reveal that co-inoculation with *T. harzianum* and *B. subtilis* produced the highest values for biomass traits under non-AMF conditions. Whereas under AMF conditions, the responses were more variable, with AMF alone or AMF combined with *T. harzianum* showing the highest values for several growth characteristics.

Root growth responses varied according to AMF status and microbial inoculation (Figure 4). Under non-AMF conditions, co-inoculation with *T. harzianum* and *B. subtilis* resulted in the highest main root length, root diameter, root volume, number of root tips, root surface area, root fresh weight, and root dry weight. This treatment also resulted in a high value of total root length, although this parameter was comparable to the uninoculated non-AMF control. Inoculation with *T. harzianum* alone under non-AMF conditions did not markedly improve root growth compared to that of the uninoculated control, except for root diameter, which was significantly increased. Similarly, *B. subtilis* produced intermediate responses, improving total root length, root tip diameter, root surface area, and root biomass relative to *T. harzianum*, but remained lower than the combined inoculation for most parameters.

Under AMF conditions, the microbial inoculants produced treatment-specific responses. *T. harzianum* significantly increased total root length, root volume, root surface area, root fresh weight, and root fresh weight, with root surface area and root dry weight reaching the highest values among all AMF treatments. In contrast, *B. subtilis* promoted total root length and root tip number, while producing intermediate values for the remaining root traits. Co-inoculation with *T. harzianum* and *B. subtilis* resulted in the greatest total root length but did not consistently outperform the individual microbial inoculations for the other root parameters.

In general, *T. harzianum* + *B. subtilis* treatment had a positive effect on the root development parameters. In comparison to other treatments, the main root’s length (28.40 cm), diameter (1.37 mm), volume (3.97 cm^3^), number of root tips per plantlet (871.80), and fresh weight (1.27 g) were all greater. Conversely, the AMF + *T. harzianum* + *B. subtilis* treatment produced the highest root length among the treatments (324.53 cm). Similarly, the AMF + *Trichoderma harzianum* treatment produced the highest root dry weight (0.37 g). These results show that under non-AMF conditions, co-inoculation with *T. harzianum* and *B. subtilis* was the most effective treatment for enhancing root morphology and biomass, whereas under AMF conditions, *T. harzianum* alone provided the greatest value in several root parameters and biomass traits.

### 2.3. Chlorophyll and Carotenoid Content

Table 1 shows the results of chlorophyll and carotenoid content of leaves estimated under various microbial treatments. Chlorophyll content was significantly affected by microbial treatments, with the highest chlorophyll a (1.89 mg·g^−1^ FW), chlorophyll b (0.60 mg·g^−1^ FW), and chlorophyll a + b (2.49 mg·g^−1^ FW) content recorded in the Control + AMF plants. Among the inoculated treatments**,** *B. subtilis* + AMF showed relatively higher chlorophyll content than the other microbial treatments. The carotenoid content in Control + AMF and *B. subtilis* + AMF treatments was higher, but these results were not statistically significant (*p* > 0.05), indicating no treatment effect on carotenoid accumulation.

### 2.4. Mineral Content

Table 2 shows the macronutrient composition from the shoots and roots of acclimated desert grass plants. In shoots, the plants grown in AMF-amended soils generally showed higher nutrient contents. In particular, the plants treated with *T. harzianum* + AMF produced the highest N concentration (3.133%), whereas Control + AMF resulted in increased accumulation of P (2.851 ppm), K (4686 ppm), and Na (320 ppm). In contrast, control plants in NAMF soil exhibited the highest Mg content (0.73%), which was statistically comparable to Control + AMF (0.78%) and *T. harzianum* + AMF (0.75%). Similarly, in roots, plants treated with AMF generally showed greater accumulation of nutrients except N and P, which showed higher concentrations in NAMF soils. For example, the highest N concentration was observed in *B. subtilis-*treated plants in NAMF soils (2.68%), and the highest P concentrations were recorded in control and *T. harzianum* + *B. subtilis-*treated plants in NAMF soils. Control + AMF plants recorded the greatest K (5361 ppm), Na (348 ppm), Ca (2.57%), and Mg (0.96%) contents. Table 3 displays the micronutrient levels estimated using the shoots and roots of micropropagated plants. The shoots treated with AMF + *B. subtilis* had the highest levels of iron (Fe, 108.74 ppm) and zinc (Zn, 88.52 ppm). In contrast, the roots had Zn and Fe concentrations of 72.38 ppm and 151.82 ppm, respectively.

### 2.5. Heatmap Correlation, Principal Component Analysis, and Hierarchical Clustering

The associations among morphological, physiological, and nutritional traits across inoculation treatments were visualized using a Pearson correlation heatmap (Figure 5). Root nutrients (RtN, RtP, RtK, RtNa, RtCa, RtMg, RtZn, and RtFe) exhibited positive correlations, suggesting coordinated nutrient uptake and accumulation within the root system. These nutrients were positively associated with most root morphological parameters, including RDW, RV, TRL, and RSA, suggesting a coordinated relationship between nutrient status and root development. Similarly, shoot nutrient concentrations (StN, StP, StK, StMg, and StCa) were positively associated with SDW and SL, indicating integrated nutrient-growth relationships in the shoot. Additionally, root development parameters were intercorrelated with leaf traits (NoLVS, LFW, LDW, LA, SD, and SFW), suggesting tightly coordinated root: shoot growth dynamics. Growth-related parameters such as NoND were positively associated with the photosynthetic pigments (Chl a, Chl b, Chl a + b, and Cart), suggesting that higher pigment concentrations are linked to increased photosynthetic activity and biomass accretion. Conversely, photosynthetic pigments and certain plant morphological parameters (NoLVS and NoND) exhibited negative associations with Na and several microelements (Zn and Fe), which may indicate nutritional competition, limited translocation, or tissue-specific accumulation patterns.

The morphological, physiological, and nutritional characteristics of the various treatments were compared using Principal Component Analysis (PCA) (Figure 6A–C). The first two components (PCs) of our data set accounted for 56% of the total variation (Figure 6A). The biplot (Figure 6B) showed that combination treatments including *T. harzianum* + *B. subtilis*, *T. harzianum* + *R. fasciculatus*, and *B. subtilis* + *R. fasciculatus* were positioned on the positive side of PC1 and were associated with improved root and shoot morphological characteristics, including RT, RSA, RDW, RFW, FW, LA, LDW, SL, SDW, and RV (Figure 6C). Among these, *T. harzianum* + *B. subtilis* were closely associated with enhanced biomass-related parameters (RDW, SDW, SFW, and LA), while *T. harzianum* + *R. fasciculatus* were associated with RtFe and RtZn. Similarly, photosynthetic pigment and certain shoot characteristics (Chl a, Chl b, Chl a + b, NoND, NoLVS) are associated with *B. subtilis* + *R. fasciculatus*. Conversely, the triple consortium (*T. harzianum* + *B. subtilis* + *R. fasciculatus*) and *R. fasciculatus* alone were positioned near the origin, indicating moderate and balanced responses across the measured parameters, whereas the NAMF, *T. harzianum*, and *B. subtilis* treatments were located on the negative side of PC1.

Hierarchical clustering clearly separated the eight inoculation treatments based on their morphological, physiological, and nutritional properties (Figure 7). Three main treatment clusters emerged from the analysis. Cluster one included non-inoculated NAMF and single inoculants (*T. harzianum* and *B. subtilis*), which were strongly associated with growth and pigment-related traits (Chl a, Chl b, Chl a + b, and carotenoids). The second cluster consisted of dual inoculants (*T. harzianum* + *B. subtilis* and *T. harzianum* + *R. fasciculatus*), which are characterized by comparatively balanced nutrient uptake, biomass accumulation, and photosynthetic pigment levels. The third cluster included single (*R. fasciculatus*), dual (*B. subtilis* + *R. fasciculatus*), and triple consortium (*T. harzianum* + *B. subtilis* + *R. fasciculatus*) treatments, which were associated with lower biomass and photosynthetic pigment accumulation but higher macronutrient (Na) and micronutrient (Zn and Fe) accumulation in root and shoot systems.

## 3. Discussion

Plant tissue culture is an essential tool for large-scale plant multiplication. However, micropropagated plants face difficulties in terms of growth, development, and survival during the acclimatization stage due to diseases caused by pathogenic microbes and harsh natural climatic conditions, resulting in a poor plant survival rate. Researchers have employed photoautotrophic micropropagation strategies, such as using ventilated culture vessels, growing plantlets under higher light and carbon dioxide conditions, and reducing the sucrose concentration in the medium, to address the transplantation issues. According to Kozai [13] and Preece and Sutter [14], these conditions facilitate healthy plant growth, leading to extensive roots and sustainable photosynthetic conditions. Alternatively, to overcome the problems of acclimatization of tissue culture plants, several researchers have reported the use of beneficial microbes such as AMF or PGPR during the hardening of in-vitro propagated plants, which is designated biological hardening [35,36,37].

Free-living soil fungi such as *Trichoderma* spp. can promote plant growth and enhance plants’ uptake of water and macronutrients. *Trichoderma* often promotes plant development by synthesizing fungal signaling metabolites, including ethylene, volatile organic compounds such as isoprenoids, and indole compounds [16]. Studies on *Zea mays*, *Lycopersicon esculentum*, and *Hordeum vlugare* plant species have shown that *Trichoderma* can significantly alter the biochemical composition of the plant’s carbohydrate, organic acid, and lipid content, improving the plant health [16]. The AMF are obligatory biotrophs that form an endosymbiotic relationship with vascular plants, thereby improving photosynthesis, micronutrient uptake, disease resistance, tolerance to drought, and temperature extremes [19]. The benefits of AMF have been demonstrated in a variety of plants, such as *Saccharum* spp. [38], *Rosa damacena* f. *trigintipetala* [24], *Hylocereus polyrhizus* [28]. The capacity of PGPR to enhance plant development and strengthen plant defense against diseases and abiotic stresses such as drought and salinity makes them crucial to agricultural output [39]. To improve the growth, development, and yield of both field-grown plants (*Zea mays*, *Peucedanum praeruptorum*) [40,41] and in vitro regenerated plants (*Nardostachys jatmansi*) [30], PGPRs such as *Bacillus* spp. and *Pseudomonas* spp. have been investigated. The combined impact of AMF and PGPR on the yield and quality of field and horticultural crops has also been studied [17,18]. In light of the aforementioned, we investigated the effects of *B. subtilis*, *R. fasciculatus*, and *T. harzianum*, singly or in combination, on the physiological, nutritional, and growth characteristics of in vitro propagated desert grass plants during acclimatization.

After ten weeks of biological establishment, the roots of desert grass plants were tested for bioinoculant colonization and sporulation. Microscopic examination revealed spores, 67% mycelium development, and 48% arbuscule formation in the roots treated with *T. harzianum*, *B. subtilis*, and *R. fasiculatus*. Germinating spores and intracellular, extracellular, and coiled hyphae were also observed in the roots of acclimated plants. These findings are consistent with *Etlingera elatior* in vitro regenerated plants treated with AMF *Gigaspora albida* and *Claroideogloumus etunicatum*, which demonstrated excellent colonization [23]. Similarly, *Philodendron bipinntifidum* micropropagated plants treated with AMF *Gigospora albida* and *G. marginata* showed 66.66% mycelium, 11.11% vesicles, and 51.22% arbuscules [27].

In vitro regenerated desert grass plants with or without AMF were used in this study to assess the effects of *T. harzianum* and *B. subtilis* separately and in combination. The results demonstrated that the combined effect of *T. harzianum* and *B. subtilis* without AMF is superior in enhancing shoot growth; the leaf area (8.38 cm^2^), leaf fresh weight (0.27 g), leaf dry weight (0.05 g), shoot length (71.21 cm), stem diameter (1.85 mm), shoot fresh weight (2.10 g), and shoot dry weight (0.49 g). Similarly, inoculation with *T. harzianum* and *B. subtilis* produced the best results for the root growth metrics, including the main root length (28.40 cm), diameter (1.37 mm), volume (3.97 cm^3^), number of root tips per plantlet (871.80), and fresh weight (1.27 g). *Trichoderma* spp., a widespread free-living soil fungus, forms a mutualistic relationship with the plants through the exchange of signaling molecules, including indole compounds, volatile organic compounds such as sesquiterpene isoprenoids, and ethylene, which modulate host biochemical and physiological processes [42]. On the other hand, plant roots release a quantitatively small but functionally significant amount of important metabolites such as lipids, carbohydrates, and amino acids, that act as chemoattractants, facilitating the establishment of beneficial interactions of plant–rhizosphere micro-organisms [43]. Once the relationship is established, these microorganisms can have a significant impact on the overall fitness of the host plant by facilitating the uptake of water and macro and micronutrients from the soil [44], protecting against harmful microorganisms and pests, and promoting overall growth and productivity. Similarly, the PGPB improves plant nutrition by fixing atmospheric nitrogen [45] or breaking down inorganic and organic sources of nutrients, such as insoluble phosphate complexes (e.g., phytate) [46]. Additionally, their co-application with *Trichoderma* works together to improve the growth of wheat [47], rice [48], and holy basil [49] plants.

The chlorophyll and carotenoid content of desert grass treated with various bioinoculants is shown in Table 1. The highest concentrations of carotenoids (0.29 mg·g^−1^ FW), chlorophyll a (1.89 mg·g^−1^ FW), chlorophyll b (0.60 mg·g^−1^ FW), and chlorophyll a + b (2.49 mg·g^−1^ FW) were found in the plants treated with AMF. These results are consistent with the earlier studies showing AMF-inoculated plants increase in chlorophyll in *Capsicum annum* [50], *Glycyrrhiza glabra* [51], *Vitis vinifera* [52], and *Philodendron bipinnatifidum* [27].

Inoculation with AMF significantly increased the accumulation of macronutrients and micronutrients in both shoot and root tissues of desert grass plants, consistent with the well-established role of AMF in nutrient uptake through extraradical hyphae networks [53,54]. Correlation analysis (Figure 5) showed positive associations between root nutrient concentration and root morphological parameters, as well as between shoot nutrient concentration and shoot growth parameters, suggesting coordinated nutrient uptake and allocation across plant organs. The multivariate analysis (PCA and hierarchical clustering) demonstrated a clear separation of treatments based on the studied parameters. It showed that the combination of *T. harzianum* + *R. fasciculatus* changes the plant’s physiological and nutritional strategy. The results indicated that the root system coordinated nutrient uptake and accumulation as the root nutrients (RtN, RtP, RtK, RtNa, RtCa, RtMg, RtZn, and RtFe) showed positive relationships. These nutrients are positively correlated with most root morphological traits, including RDW, RV, TRL, and RSA, suggesting that improved nutrient uptake fosters root growth and plant vigor. Nutrient-regulating shoot growth was also demonstrated by positive associations between shoot nutrient concentrations, such as StN, StP, StK, StMg, and StCa, and SDW and SL. Furthermore, NoLVS, leaf biomass (LFW and LDW), LA, SD, and SFW are intercorrelated with root development parameters, indicating tightly coordinated root: shoot growth. The symbiosis of AMF with crop plants has been shown by Nadeem et al. [53] and Turrini et al. [54] to improve nutrient uptake, including P, N, S, K, Ca, Cu, and Zn, which promotes host plant growth and development. Furthermore, *Trichoderma* enhanced the uptake of macronutrients (Ca, Mg, P, N, K, and S) and micronutrients (Cu, Fe, Mn, Mo, and Zn) and improved host plant development, according to Yeddia et al. [55] and Ramakrishna et al. [44]. In a similar vein, *Trichoderma asperellum* encouraged plant mineralization and biomass growth in tobacco, according to Singh et al. [56]. Furthermore, it has been demonstrated that *Trichoderma* species contribute to the solubilization of soil nutrients that are taken up by the roots of host plants, including metallic Zn, MnO_2_, and rock phosphate, primarily Ca_3_(PO_3_)_2_ [57,58]. Overall, these results demonstrate that targeted microbial inoculants, when used in strategic combinations, provide significant physiological benefits during the acclimatization of micropropagated plants, thus supporting their application in ecological restoration programs.

## 4. Materials and Methods

### 4.1. Study Location and Plant Material

This research was carried out at the plant tissue culture facility of King Saud University’s College of Food and Agricultural Sciences. In vitro shoots of *Panicum turgidum* Forssk. were replicated on Murashige and Skoog’s medium (MS) [59], which included 30 g/L sucrose and 0.5 mg/L of thidiazuron (TDZ). The medium was autoclaved for 15 min at 121 °C and 103 kPa after the addition of 0.8% agar-agar (Duchefa Biochemie, Haarlem, The Netherlands) and after adjusting its pH to 5.8. The cultures were maintained at an air temperature of 25 ± 2 °C, with a photosynthetic photon flux density (PPFD) of 25 µmol m^−2^·s^−1^ supplied by cool white, fluorescent tubes, under a 16/8 light/dark photoperiod for four weeks. The proliferated axillary shoots (Figure 8a), measuring 4–5 cm, were individually cultured onto MS medium supplemented with indole-3-butyric acid (IBA) at a concentration of 2 mg/L for their rooting (Figure 8b). Following three weeks of culture, the plantlets were carefully removed from the gelled medium, rinsed with tap water, and used as plant materials for acclimatization experiments in this study.

### 4.2. Preparation and Inoculation of Bioinoculants

The *R. fasciculatus* (AMF) with 33.4 spores/gram of dry soil, *T. harzianum* (Figure 9a), and *B. subtilis* (Figure 9b) were used as inoculum. *B. subtilis* was cultured in conical flasks (250 mL) that contained 100 mL of nutrient broth medium. The flasks were then incubated on a rotary shaker at 200 rpm for 72 h at 28 ± 2 °C. Later, the bacterial suspensions were diluted with water to produce 1 × 10^8^ colony-forming units (CFU) mL^−1^. *Trichoderma harzianum* was cultivated at 28 °C under dark conditions, onto Potato Sucrose Agar (PSA) plates composed of 200 g/L potato, 15 g/L sucrose, 20 g/L agar-agar, until the colony showed a green coloration. Spores were subsequently transferred using sterile distilled water to the crystallizers, achieving a concentration of 10^7^ spores mL^−1^ in the conidial suspension [60]. The plants were cultured in plastic pots measuring 21.5 cm in length, 4 cm in height, and 1.7 cm in width, filled with a sterile sand–soil mixture (1:1; *v*/*v*). The potted plants were grown for 10 weeks in a controlled environment at 25 ± 2 °C, with a relative humidity (RH) maintained at 50–60% and a PPFD of 100 µmol m^−2^·s^−1^, under a 16:8 h of light/dark photoperiod provided by white fluorescent lamps. The cultures received regular irrigation with a phosphorus-free Hoagland’s nutrition solution. The experiments were set up in a factorial complete randomized design. One factor involved the AMF (Figure 9c,d) treatments, no-AMF (NAMF) and AMF (5%; *w*/*w*), while the other factor comprised four levels of microbial inoculations (Figure 1): (1) control, (2) *T. harzianum*, (3) *B. subtilis*, and (4) combination of *T. harzianum* and *B. subtilis*. Each treatment included 50 replicates, and each replicate was represented by a pot containing one plantlet.

### 4.3. Estimation of Symbiotic Root Development and AMF Spore Count

The percentage of root colonization was quantified by selecting, staining, and analyzing fresh fine roots of *P. turgidum* plantlets, in accordance with the methods established by Phillips and Hayman [61]. The roots underwent a cleaning process and were subsequently treated with 10% KOH at 80 °C for 30 min. After washing, the samples were treated with 3% H_2_O_2_ for 3 min, subsequently acidified with 1% HCl for 10 min, and finally stained with Trypan blue at 80 °C for an additional 20 min. Later, from each stained root sample, 50 segments were selected and mounted on a glass slide (25 segments/slide) using a lactoglycerol solution. The coverslip was precisely placed to analyze different structures, such as mycelium, vesicles, and arbuscules, in the root segments using an optical microscope at 400× magnification. Spores were isolated from the substrate of each treatment following the methods established by Gerdemann and Nicolson [62], and the spore population for each treatment was determined per 100 g of dry soil.

### 4.4. Measurements of Vegetative Growth Parameters

The plants were harvested following 10 weeks of cultivation, and growth parameters, including shoot and root length (cm), number of leaves, number of nodes, and stem diameter (mm), were recorded. Shoot and root dry weights (g) were measured following a 48-h oven-drying period at 70 °C. The leaf area was determined using a portable area meter (CI-202; CID, Inc., Vancouver, WA, USA). The roots of *P. turgidum* plantlets from each treatment were extracted from the pots and rinsed with tap water to create root replicates for five plants. The toluidine red staining procedure of roots was carried out according to the protocol of Veiga et al. [63] and Retamales and Scharaschkin [64]. The roots were dyed with toluidine red for approximately 8 h before scanning. A flatbed scanner (Canon unit 101, Green Island, NY, USA) was used for the scanning. The total root length (cm), root surface area (cm^2^), root diameter (mm), root volume (cm^3^), and number of root tips were recorded using WinRHIZO software (v5.0, Regent Instruments, Quebec, QC, Canada) for the scanned roots.

### 4.5. Measurements of Plant Pigments

The contents of chlorophyll a, chlorophyll b, and total carotenoids were determined by homogenizing 50 mg of fresh plant leaf tissue in 10 mL of 80% acetone solution [65]. The homogenized samples were centrifuged at 5000 rpm for 5 min using a Benchtop Centrifuge 5810R (Eppendorf, Hamburg, Germany). The resulting supernatants were incubated in the dark for three hours before absorbance was measured using a UV–VIS spectrophotometer (UV1800, SHIMADZU, Kyoto, Japan). Absorbance readings were recorded at 663 nm for chlorophyll a, 646 nm for chlorophyll b, and 470 nm for total carotenoids. The concentrations of chlorophyll a, chlorophyll b, and carotenoids were calculated using the following equations.

Chl a (mg g^−1^) = (12.72 × OD663 − 2.5 × OD646) V/1000 W

Chl b (mg g^−1^) = (22.88 × OD646 − 4.67 × OD663) V/1000 W

where V is the total volume of acetone extract (mL), and W is the FW (G) of the sample.

### 4.6. Estimation of Plant Mineral Content

The shoot and root parts of *P. turgidum* were oven-dried for 48 h at 70 °C and ground separately into a powder. Half a gram of oven-dried powder material from each sample was placed in the digestive vessels with 10 mL of concentrated H_2_SO_4_. Subsequently, the samples were digested at 200 °C for 2 h using the microwave block digestor MDS-6G (SINEO Microwave Chemistry Technology, Shanghai, China). To clear the solution, 3 drops of H_2_O_2_ were carefully added after the digested materials had cooled to room temperature. The digests were filtered through Whatman No. 42 filter paper after being diluted with distilled water to a final volume of 50 mL. Total nitrogen (N) was determined by Kjeldahl’s method [66] using an automatic Kjeldahl distillation unit (K9840; Hannon Instruments, Shanghai, China). Potassium (K), Sodium (Na), and Calcium (Ca) were determined using a flame photometer (Jenway PFP-7, Bibby Scientific Ltd., Staffordshire, UK) following the method described in Chapman and Pratt [67]. The concentrations of Magnesium (Mg), Zinc (Zn), and Iron (Fe) in the prepared samples were determined using an atomic absorption spectrophotometer (Hitachi Polarized Zeeman AAS, Z-8200, Hitachi, Japan) according to AOAC [68].

### 4.7. Statistical Analysis

The dataset was statistically analyzed using the SAS program (Version 9.4; SAS Institute, Inc., Cary, NC, USA) with an analysis of variance (ANOVA) for factorial design. Upon significance of the mean values, Tukey’s honest difference (HSD) (*p* = 0.05) was used to determine the significant difference among the means of treatments. Furthermore, multivariate analyses such as Pearson’s correlation heatmap, Principal component analysis (PCA), and hierarchical clustering were used to assess the effects of inoculation treatments on the morphological, photosynthetic, and nutrient-uptake characteristics of the target plant using JMP ^®^ 16.0.0. Cluster analysis.

## 5. Conclusions

This study provides new insights into the morphological, physiological, and nutritional responses of control and treated desert grass tissue-cultured plants during their acclimatization stage with bioinoculants *R. fasciculatus*, *B. subtilis*, and *T. harzianum*. Plants treated with bioinoculants showed improved shoot and root growth parameters, chlorophyll and carotenoid content, and nutrient uptake, including macro- and micronutrients. Growth and physiological characteristics showed a correlation with mineral nutrient absorption in plants treated with the inoculant combination. Based on these findings, we conclude that supplementation of useful bioinoculants such as *R. fasciculatus*, *B. subtilis*, and *T. harzianum* is beneficial during acclimatization of tissue-cultured plants.

## Figures and Tables

**Figure 1:**
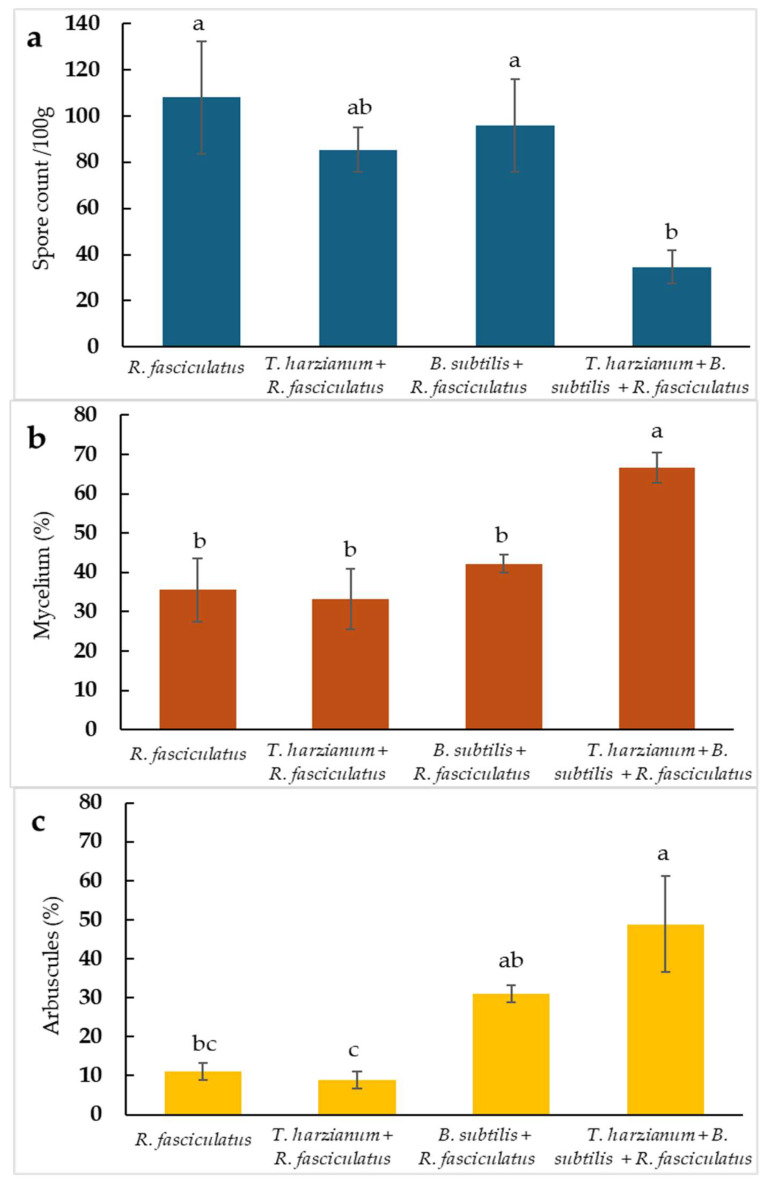
Spore count (**a**), mycelium percentage (**b**), and arbuscule percentage (**c**) in the roots of micropropagated *Panicum turgidum* plantlets in response to *Rhizophagus fasciculatus*, *Trichoderma harzianum*, and *Bacillus subtilis* treatments following 10 weeks of acclimatization. Values followed by the same letter are not significantly different at the *p* ≤ 0.05 level, according to Tukey’s multiple range test.

**Figure 2:**
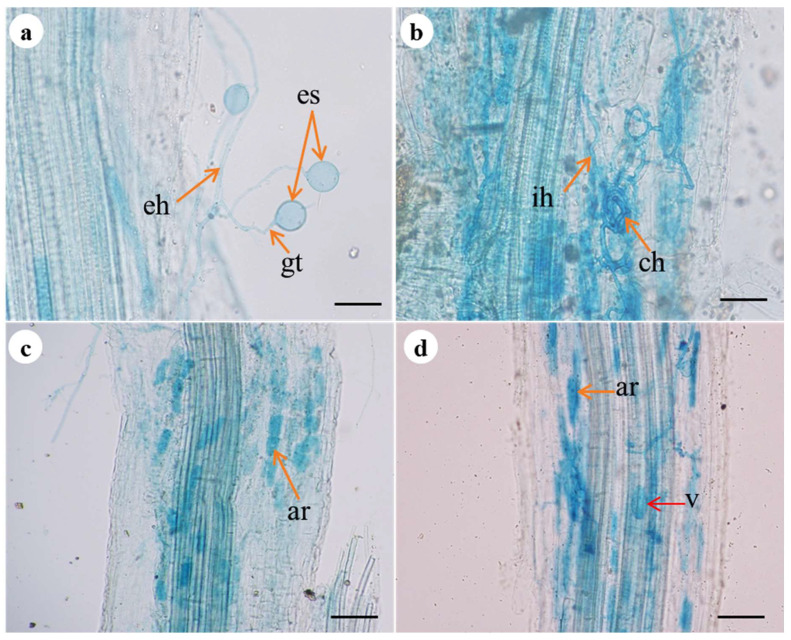
Photomicrographs indicating the abundance of arbuscular mycorrhizal colonization in the roots of micropropagated *Panicum turgidum* plantlets during acclimatization. (**a**–**d**) Extraradical intact spores (es); intercellular hyphae (ih); arbuscules (Ar); extracellular hyphae (eh); coiled hyphae (ch); vesicles (v); and germination tube (gt).

**Figure 3:**
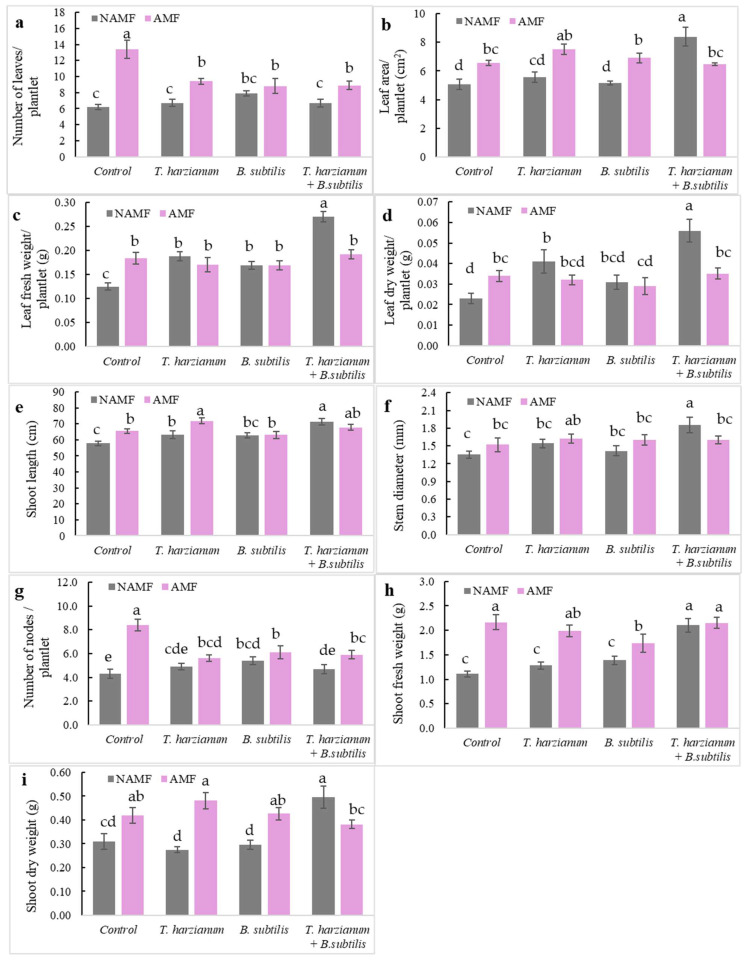
Vegetative growth characteristics of micropropagated *Panicum turgidum* plantlets in response to *Rhizophagus fasciculatus*, *Trichoderma harzianum*, and *Bacillus subtilis* treatments following 10 weeks of acclimatization. (**a**) Number of leaves per plantlet, (**b**) Leaf area per plantlet, (**c**) Leaf fresh weight per plantlet, (**d**) Leaf dry weight per plantlet, (**e**) Shoot length, (**f**) Stem diameter, (**g**) number of nodes per plantlet, (**h**) Shoot fresh weight, and (**i**) Shoot dry weight. Values followed by the same letter are not significantly different at the *p* ≤ 0.05 level, according to Tukey’s multiple range test.

**Figure 4:**
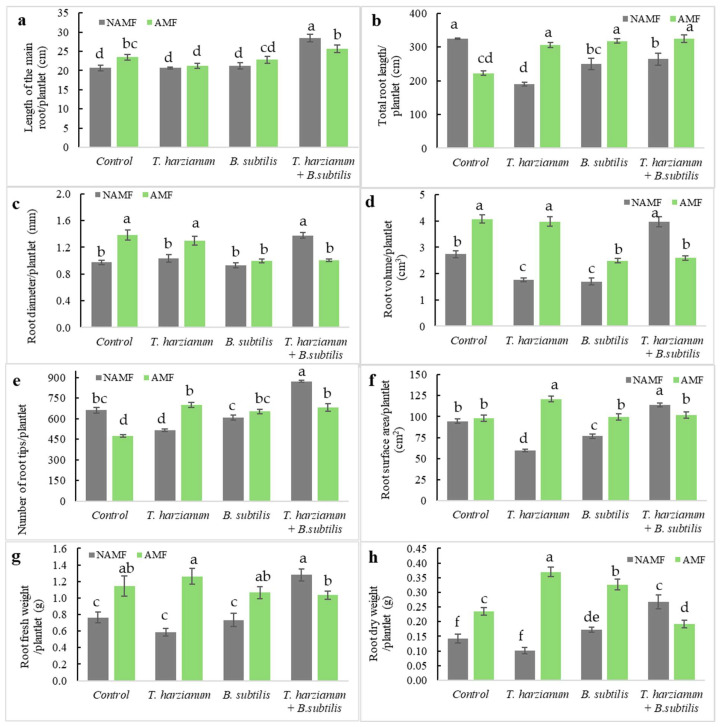
Root growth characteristics of micropropagated *Panicum turgidum* plantlets in response to *Rhizophagus fasciculatus*, *Trichoderma harzianum*, and *Bacillus subtilis* treatments following 10 weeks of acclimatization. (**a**) Length of the main root per plantlet, (**b**) total root length per plantlet, (**c**) root diameter per plantlet, (**d**) root volume per plantlet, (**e**) number of root tips per plantlet, (**f**) root surface area per plantlet, (**g**) root fresh weight per plantlet, and (**h**) root dry weight per plantlet. Values followed by the same letter are not significantly different at the *p* ≤ 0.05 level, according to Tukey’s multiple range test.

**Figure 5:**
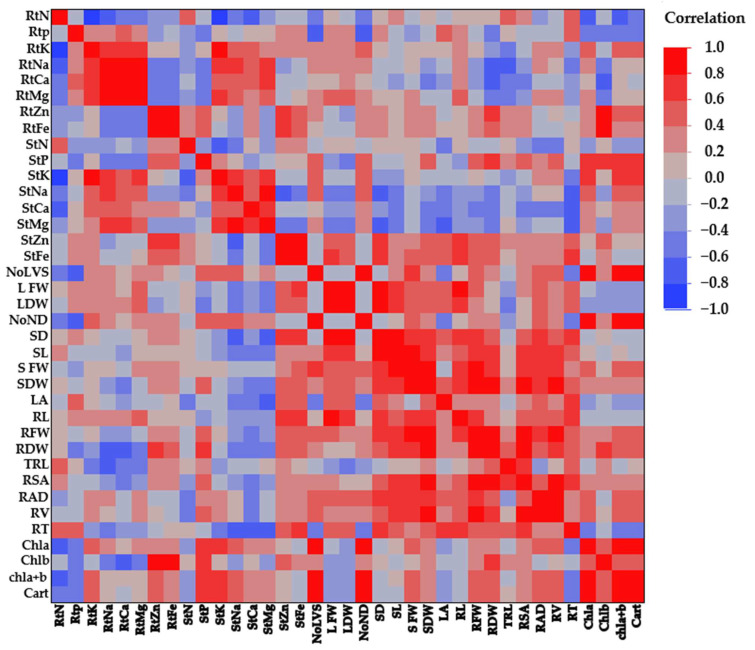
A correlation heatmap of the relationship between morphological, physiological, and nutritional parameters. Red colors indicate a strong positive correlation, while blue indicates a significant negative correlation. The abbreviations RtN: Root Nitrogen, RtP: Root Phosphorus, RtK: Root Potassium, RtNa: Root Sodium, RtCa: Root Calcium, RtMg: Root Magnesium, RtZn: Root Zinc RtFe: Root Iron, StN: Shoot Nitrogen StP: Shoot Phosphorus, StK: Shoot Potassium, StCa: Shoot Calcium, StMg: Shoot Magnesium, StZn: Shoot Zinc, StFe: Shoot Iron, NoLVS: Number of Leaves, LFW: Leaf Fresh Weight, LDW: Leaf Dry Weight, NoND: Number of Nodes SD: Stem Diameter, SL: Shoot Length, SFW: Shoot Fresh Weight, SDW: Shoot Dry Weight, LA: Leaf Area, RL: Root Length, RFW: Root Fresh Weight RDW: Root Dry Weight, TRL, Total Root Length, RSA: Root Surface Area, RAD: Root Average Diameter, RV: Root Volume, RT: Root Tips Chl a: Chlorophyll a, Chl b: Chlorophyll b, Chl a + b: Total Chlorophyll Cart: Carotenoids.

**Figure 6:**
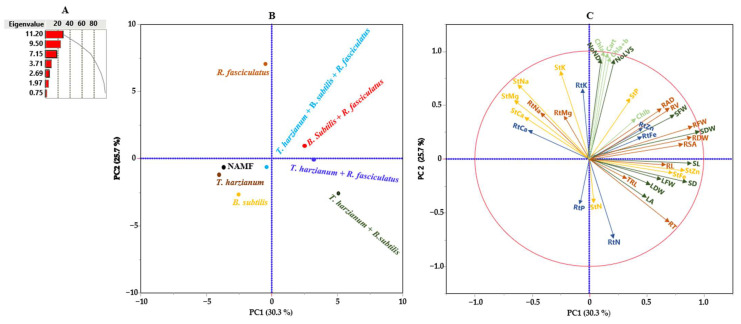
Principal Component Analysis (PCA) showing the relationship between morphological, physiological, and nutritional parameters. (**A**) showing eigenvalues of components, (**B**) showing the treatment along PC1 and PC2, and (**C**) showing the study’s parameters. The abbreviations RtN: Root Nitrogen, RtP: Root Phosphorus, RtK: Root Potassium, RtNa: Root Sodium, RtCa: Root Calcium, RtMg: Root Magnesium, RtZn: Root Zinc RtFe: Root Iron, StN: Shoot Nitrogen StP: Shoot Phosphorus, StK: Shoot Potassium, StCa: Shoot Calcium, StMg: Shoot Magnesium, StZn: Shoot Zinc, StFe: Shoot Iron, NoLVS: Number of Leaves, LFW: Leaf Fresh Weight, LDW: Leaf Dry Weight, NoND: Number of Nodes SD: Stem Diameter, SL: Shoot Length, SFW: Shoot Fresh Weight, SDW: Shoot Dry Weight, LA: Leaf Area, RL: Root Length, RFW: Root Fresh Weight RDW: Root Dry Weight, TRL, Total Root Length, RSA: Root Surface Area, RAD: Root Average Diameter, RV: Root Volume, RT: Root Tips Chl a: Chlorophyll a, Chl b: Chlorophyll b, Chl a + b: Total Chlorophyll Cart: Carotenoids.

**Figure 7:**
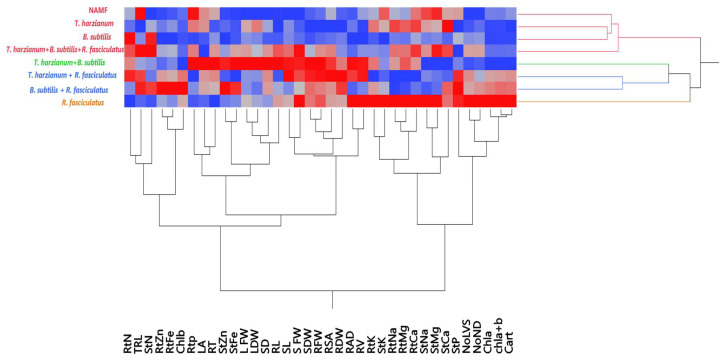
Hierarchical clustering heatmap analysis between morphological, physiological, and nutritional parameters. The abbreviations RtN: Root Nitrogen, RtP: Root Phosphorus, RtK: Root Potassium, RtNa: Root Sodium, RtCa: Root Calcium, RtMg: Root Magnesium, RtZn: Root Zinc RtFe: Root Iron, StN: Shoot Nitrogen, StP: Shoot Phosphorus, StK: Shoot Potassium, StCa: Shoot Calcium, StMg: Shoot Magnesium, StZn: Shoot Zinc, StFe: Shoot Iron, NoLVS: Number of Leaves, LFW: Leaf Fresh Weight, LDW: Leaf Dry Weight, NoND: Number of Nodes SD: Stem Diameter, SL: Shoot Length, SFW: Shoot Fresh Weight, SDW: Shoot Dry Weight, LA: Leaf Area, RL: Root Length, RFW: Root Fresh Weight RDW: Root Dry Weight, TRL, Total Root Length, RSA: Root Surface Area, RAD: Root Average Diameter, RV: Root Volume, RT: Root Tips Chl a: Chlorophyll a, Chl b: Chlorophyll b, Chl a + b: Total Chlorophyll, Cart: Carotenoids. Red color indicates relatively high values, while blue color indicates relatively low values for each treatment.

**Figure 8:**
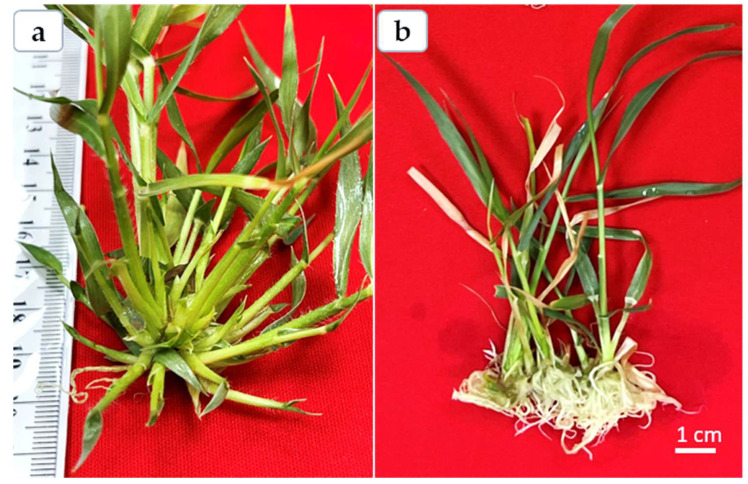
Axillary shoots of *P. turgidum* multiplied onto MS medium containing 0.5 mg/L thidiazuron following 4 weeks in culture (**a**), and rooted onto MS medium containing 2 mg/L indole-3-butyric acid following 3 weeks in culture (**b**).

**Figure 9:**
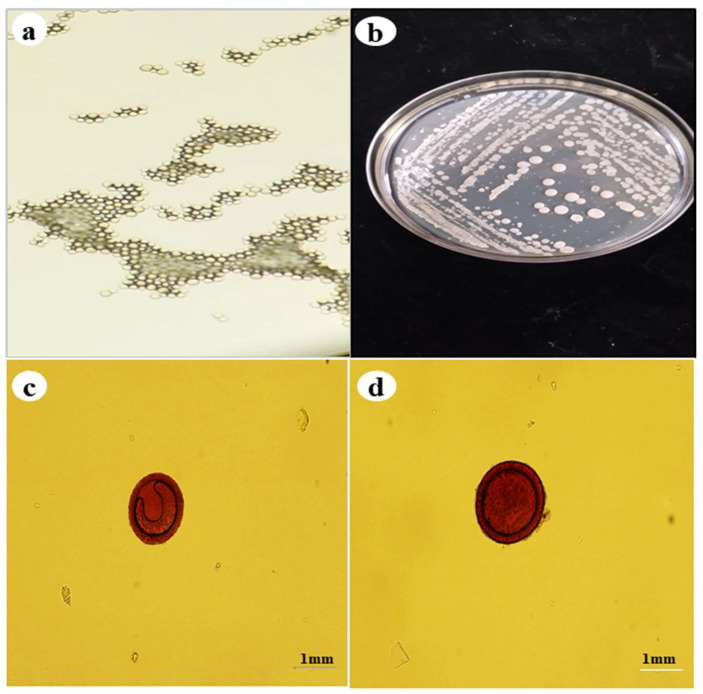
Spores of (**a**) *Trichoderma harzianum*, (**b**) *Bacillus subtilis* strain, and (**c**,**d**) intact spores of arbuscular mycorrhizal fungus *Rhizophagus fasciculatus*.

**Table 1:** Content of chlorophyll and carotenoid pigments in micropropagated *Panicum turgidum* plantlets in response to *Rhizophagus fasciculatus*, *Trichoderma harzianum*, and *Bacillus subtilis* treatments fol-lowing 10 weeks of acclimatization.

Treatments	Status	Chlorophyll a (mg·g^−1^FW)	Chlorophyll b (mg·g^−1^FW)	Chlorophyll a + b (mg·g^−1^FW)	Carotenoids (mg·g^−1^FW)
Control	NAMF	1.25 ± 0.27 cd	0.34 ± 0.08 bc	1.60 ± 0.19 abc	0.22 ± 0.08 ^NS^
	AMF	1.89 ± 0.03 a	0.60 ± 0.06 a	2.49 ± 0.09 a	0.29 ± 0.02
*Trichoderma harzianum*	NAMF	1.06 ± 0.25 cd	0.33 ± 0.06 bc	1.39 ± 0.19 bc	0.17 ± 0.07
	AMF	1.49 ± 0.19 bc	0.41 ± 0.05 bc	1.90 ± 0.15 abc	0.26 ± 0.06
*Bacillus subtilis*	NAMF	1.06 ± 0.24 d	0.33 ± 0.06 bc	1.39 ± 0.18 bc	0.18 ± 0.08
	AMF	1.68 ± 0.23 ab	0.50 ± 0.04 ab	2.18 ± 0.19 ab	0.29 ± 0.07
*T. harzianum* + *B. subtilis*	NAMF	1.03 ± 0.32 d	0.32 ± 0.08 bc	1.35 ± 0.25 c	0.17 ± 0.08
	AMF	1.20 ± 0.07 cd	0.34 ± 0.02 bc	1.53 ± 0.05 bc	0.20 ± 0.03
*p*-values					
Treatments		0.0078 **	0.0060 **	0.0001 ***	0.1060 ^NS^
Status (NAMF-AMF)		0.2286 ^NS^	0.1452 ^NS^	0.0189 *	0.7295 ^NS^
Treatments × Status		0.6759 ^NS^	0.2382 ^NS^	0.1953 ^NS^	0.9228 ^NS^

Different letters within a set of values in each column denote significant differences at *p* ≤ 0.05 according to Tukey’s multiple range test. NS = non-significant. *, ** and *** = Significant at *p* ≤ 0.05, *p* ≤ 0.01 and *p* ≤ 0.001, respectively. AMF = *Rhizophagus fasciculatus* arbuscular mycorrhizal fungi inoculation. NAMF = non-treated with AMF.

**Table 2:** Macro-mineral content in the shoot and root of micropropagated *Panicum turgidum* plants in re-sponse to *Rhizophagus fasciculatus*, *Trichoderma harzianum*, and *Bacillus subtilis* treatments following 10 weeks of acclimatization.

Treatments		N (%)	P (ppm)		K (ppm)		Na (ppm)	Ca (%)	Mg (%)
Shoot nutrients									
Control	NAMF	1.077 ± 0.06 d	2.589 ± 0.27 ab	3673 ± 286.45 b		268 ± 18.48 b		2.09 ± 0.12 c	0.73 ± 0.07 a
	AMF	1.273 ± 0.08 cd	2.851 ± 0.19 a	4686 ± 413.29 a		320 ± 5.36 a		2.44 ± 0.22 bc	0.78 ± 0.06 a
*Trichoderma harzianum*	NAMF	2.034 ± 0.12 b	2.155 ± 0.15 b	2783 ± 266.01 c		198 ± 10.84 c		3.21 ± 0.30 a	0.52 ± 0.05 b
	AMF	1.625 ± 0.09 bc	2.834 ± 0.23 a	1799 ± 145.72 de		152 ± 10.91 d		1.18 ± 0.10 d	0.36 ± 0.03 c
*Bacillus subtilis*	NAMF	2.982 ± 0.16 a	2.542 ± 0.21 ab	1350 ± 107.86 e		146 ± 7.81 d		1.19 ± 0.09 d	0.41 ± 0.04 bc
	AMF	2.788 ± 0.18 a	2.903 ± 0.19 a	2651 ± 208.73 c		110 ± 6.64 e		2.55 ± 0.19 bc	0.34 ± 0.03 cd
*T. harzianum* + *B. subtilis*	NAMF	1.855 ± 0.15 b	2.395 ± 0.22 ab	2340 ± 213.40 cd		85 ± 6.08 e		0.72 ± 0.07 d	0.20 ± 0.02 d
	AMF	3.133 ± 0.32 a	2.150 ± 0.17 b	2233 ± 220.55 cd		207 ± 14.29 c		2.74 ± 0.18 ab	0.75 ± 0.07 a
*p*-values									
Treatments		0.0000 ***	0.1262 ^NS^	0.0000 ***		0.0000 ***		0.0211 *	0.0000 ***
Status		0.0819 ^NS^	0.0850 ^NS^	0.1012 ^NS^		0.0086 **		0.0034 **	0.0157 *
Treatments × Status		0.0006 ***	0.1971 ^NS^	0.0010 ***		0.0000 ***		0.0000 ***	0.0000 ***
Root nutrients									
Control	NAMF	1.57 ± 0.10 de	2.836 ± 0.27 a	3110 ± 284.80 c		198 ± 11.72 d		2.07 ± 0.15 bc	0.56 ± 0.04 c
	AMF	0.28 ± 0.02 g	1.868 ± 0.15 a	5361 ± 307.83 a		348 ± 18.58 a		2.57 ± 0.18 a	0.96 ± 0.09 a
*Trichoderma harzianum*	NAMF	0.92 ± 0.06 f	2.440 ± 0.13 ab	3870 ± 304.91 b		295 ± 16.29 b		2.32 ± 0.17 ab	0.77 ± 0.06 b
	AMF	2.51 ± 0.17 ab	1.746 ± 0.14 a	1398 ± 109.31 d		78 ± 6.56 f		0.55 ± 0.03 e	0.27 ± 0.02 e
*Bacillus subtilis*	NAMF	2.68 ± 0.16 a	1.797 ± 0.17 ab	1883 ± 146.72 d		150 ± 8.62 e		1.71 ± 0.10 c	0.44 ± 0.04 cd
	AMF	1.25 ± 0.09 ef	2.111 ± 0.16 bc	3445 ± 212.99 bc		82 ± 5.69 f		1.20 ± 0.10 cd	0.32 ± 0.02 de
*T. harzianum* + *B. subtilis*	NAMF	1.91 ± 0.16 cd	2.860 ± 0.23 ab	3870 ± 263.76 b		221 ± 11.79 cd		1.95 ± 0.17 bc	0.78 ± 0.05 b
	AMF	2.24 ± 0.21 bc	2.423 ± 0.15 ab	2722 ± 270.88 c		252 ± 14.18 c		2.37 ± 0.14 ab	0.72 ± 0.05 b
*p*-values									
Treatments		0.0000 ***	0.0084 **	0.0000 ***		0.0000 ***		0.0000 ***	0.0000 ***
Status		0.0523 ^NS^	0.0032 **	0.7868 ^NS^		0.0093 **		0.0026 **	0.0681 ^NS^
Treatments × Status		0.0000 ***	0.0172 *	0.0000 ***		0.0000 ***		0.0000 ***	0.0000 ***

Different letters within a set of values in each column denote significant differences at *p* ≤ 0.05 according to Tukey’s multiple range test. NS = non-significant. *, ** and *** = Significant at *p* ≤ 0.05, *p* ≤ 0.01 and *p* ≤ 0.001, respectively.

**Table 3:** Micro-mineral content in the shoot and root of micropropagated *Panicum turgidum* plants in response to *Rhizophagus fasciculatus*, *Trichoderma harzianum*, and *Bacillus subtilis* treatments following 10 weeks of acclimatization.

Treatments		Zn (ppm)	Fe (ppm)
Shoot nutrients			
Control	NAMF	28.57 ± 2.95 cd	51.80 ± 4.81 c
	AMF	38.15 ± 3.12 bc	65.57 ± 3.58 bc
*Trichoderma harzianum*	NAMF	35.08 ± 3.21 bcd	55.56 ± 3.43 c
	AMF	32.05 ± 2.99 cd	51.02 ± 4.43 c
*Bacillus subtilis*	NAMF	26.7 ± 2.21 d	56.22 ± 5.09 c
	AMF	88.52 ± 4.66 a	108.74 ± 6.57 a
*T. harzianum* + *B. subtilis*	NAMF	83.10 ± 5.30 a	119.93 ± 6.35 a
	AMF	43.65 ± 3.81 b	76.29 ± 5.47 b
*p*-values			
Treatments		0.0000 ***	0.0000 ***
Status		0.0129 *	0.2256 ^NS^
Treatments × Status		0.0000 ***	0.0000 ***
Root nutrients			
Control	NAMF	20.33 ± 1.76 cd	39.31 ± 2.40 d
	AMF	31.25 ± 2.00 bc	55.32 ± 3.00 bc
*Trichoderma harzianum*	NAMF	26.59 ± 2.30 bcd	57.33 ± 4.10 cd
	AMF	36.75 ± 2.58 cd	69.21 ± 4.45 b
*Bacillus subtilis*	NAMF	18.85 ± 1.57 d	31.71 ± 2.40 d
	AMF	72.38 ± 6.40 a	151.82 ± 8.44 a
*T. harzianum* + *B. subtilis*	NAMF	24.56 ± 2.02 a	54.18 ± 3.24 cd
	AMF	31.67 ± 2.70 b	64.10 ± 4.18 bc
*p*-values			
Treatments		0.0000 ***	0.0000 ***
Status		0.0000 ***	0.0000 ***
Treatments × Status		0.0000 ***	0.0000 ***

Different letters within a set of values in each column denote significant differences at *p* ≤ 0.05 according to Tukey’s multiple range test. * = Significant at *p* ≤ 0.05; *** = Significant at *p* ≤ 0.001.

## Data Availability

Data and materials supporting the results or analyses presented in the article are available upon reasonable request.
